# Comparative evaluation of biphasic calcium phosphate and biphasic calcium phosphate collagen composite on osteoconductive potency in rabbit calvarial defect

**DOI:** 10.1186/s40824-014-0026-7

**Published:** 2015-02-12

**Authors:** Eun-Ung Lee, Dong-Ju Kim, Hyun-Chang Lim, Jung-Seok Lee, Ui-Won Jung, Seong-Ho Choi

**Affiliations:** Department of Periodontology, Research Institute for Periodontal Regeneration, College of Dentistry, Yonsei University, 50 Yonsei-ro, Seodaemun-gu, Seoul South Korea

**Keywords:** Biphasic calcium phosphate, Bone regeneration, Bone substitutes, Collagen, Osteoconduction

## Abstract

**Background:**

The aim of this study was to determine the osteoconductivity of biphasic calcium phosphate collagen composite (BCPC) in rabbit calvarial defect model by comparing with biphasic calcium phosphate (BCP). Four 8 mm diameter bicortical calvarial defects were made in ten rabbits. Each of the defects was randomly assigned and filled with 1) collagen sponge, 2) BCP, 3) BCPC, and 4) nothing as control. The animals were sacrificed at either 2 weeks (n = 5) or 8 weeks (n = 5) healing period.

**Results:**

All groups showed wedge shaped new bone formation limited to the area of the defect margin at both healing periods. The amounts of new bone and defect closure were similar among all groups. In the control and collagen sponge group, the center of the defect was depressed by surrounding tissues. In contrast, in BCP and BCPC group, the center of the defect did not depressed and the grafted materials maintained the space. And the augmented area was significantly higher in BCP and BCPC group compared to the control and collagen sponge group at both healing periods (p < 0.05).

**Conclusions:**

The BCPC and BCP demonstrated proper space maintaining capacity and osteoconductive property, suggesting BCPC can be efficiently utilized in various clinical situations.

## Background

Various bone graft materials have been developed and used for reconstruction of bony defects in periodontal and implant surgeries. The autogenous bone is considered as the gold standard because of its superior capacity in bone formation, however, has some clinical drawbacks such as a morbidity of donor site and uncontrolled resorption rate [1,2]. Therefore, there have been many attempts to develop bone graft materials which can replace and substitute the autogenous bone.

Among various bone graft materials, calcium phosphate (Ca-P) bone substitutes such as hydroxyapatite (HA) and beta-tricalcium phosphate (β-TCP) have been widely used because their chemical and structural characters are similar to those of human bone [1]. Indeed, they have shown favorable biocompatibility and osteoconductivity when used as bone graft materials [3]. Among Ca-Ps, HA, which is very stable, can maintain the space effectively but has low osteoconductivity [4,5]. In contrast, β-TCP is more biodegradable and rapidly replaced by newly formed bone but has low capacity of space maintaining [6]. Therefore biphasic calcium phosphate (BCP), which is composed of HA and β-TCP, was introduced to overcome limitations of each material and several studies have been demonstrated that BCPs can be used as bone substitutes successfully [5,7,8].

Particle type BCPs have been frequently used and their clinical efficacy was well-demonstrated. However, there are some limitations on using particle type BCPs for bone substitutes. Particle type BCPs are susceptible to external compressive forces if not protected properly and easy to be collapsed in non-contained defects [9]. Therefore, the use of particle type BCPs may give technical difficulties to clinicians in specific situations. Hence, block type BCPs have been designed to impose stability in the defect of unfavorable. However, the application of block type BCPs requires adaptation, trimming and securement, which may increase possibilities of surgical error. Moreover, bone in-growth into the bone substitutes of block type depends much upon its structural properties, and some studies reported limited bone formation even though they were used with osteoinductive materials [9,10].

Recently, BCP collagen composite (BCPC) graft materials have been developed and explored as bone substitutes. Due to excellent flexibility of the collagen, BCPC can be easily molded into the desired shape while maintaining the initial shape [11]. Moreover, collagen can promote healing process by retaining blood clots and inducing migration of fibroblasts [12]. Therefore BCPC can be expected to have more favorable biological and mechanical characteristics for bone formation and clinical handling, compared with above described BCPs. Thus, the aim of this study was to determine the osteoconductivity of BCPC in rabbit calvarial defect model by comparing with BCP.

## Methods

### Materials

The collagen sponges, BCP and BCPC were used as graft materials in this study. The collagen sponges were made of bovine type I collagen with cylinder form (8 mm diameter and 2 mm vertical height). The BCP (Osteon™ II, Genoss. Co. Ltd, Suwon, South Korea), a particle type graft material, was composed of HA and β-TCP with the weight ratio of 30/70. The sizes of particle and pore were 0.5 mm to 1.0 mm and 250 μm respectively and the porosity was 70%. The BCPC (Osteon™ II collagen, Genoss. Co. Ltd, Suwon, South Korea) was block type graft material with cylinder form (8 mm diameter and 2 mm vertical height). It was composed of BCP (Osteon™ II, Genoss. Co. Ltd, Suwon, South Korea) and the bovine type I collagen and the weight percentage of the collagen was 4%.

### Animals

Ten male New-Zealand white rabbits (mean body weight, 3.0 kg to 3.5 kg; 14 weeks to 16 weeks old) were used. All animals were housed in separate cages with standard laboratory conditions and fed with standard diets. All of the procedures including animal selection, management, preparation and surgical procedures followed protocols approved by the Institutional Animal Care and Use Committee, Yonsei Medical Center, Seoul, Korea (approval number 2012–0031).

### Study design

**Figure 1:**
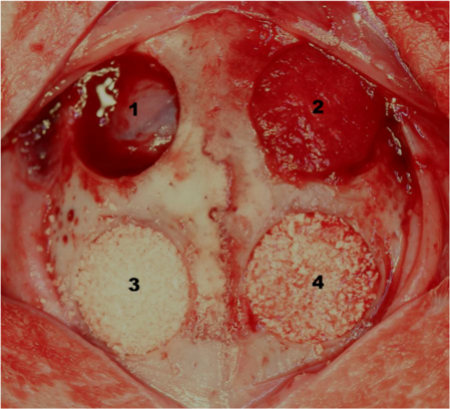
**Four circular defects with a diameter of 8 mm were made in the rabbit calvarium and the experimental materials were grafted as follows: (1) no graft, (2) Collagen sponge, (3) BCP, (4) BCPC.**

In each rabbit calvarium, four circular bi-cortical defects with a diameter of 8 mm were formed. And then, the defects were randomly assigned to one of the following four experimental groups and filled with each material (Figure 1). The rabbits were allowed to get healing period of 2 weeks (n = 5) or 8 weeks (n = 5).

- Control group: The defect was filled with blood clot only.
- Collagen group: The collagen sponge was filled in the defect.
- BCP group: BCP was filled in the defect.
- BCPC group: BCPC was filled in the defect.

### Surgical procedure

The animals were intramuscularly injected and anesthetized with a mixture of ketamine hydrochloride (Ketalar VR, Yuhan Co, Seoul, Korea) and xylazine (Rumpun VR, Bayer Korea Ltd, Seoul, Korea). After that, the surgical sites were shaved and draped with povidone iodine and alcohol followed by local anesthesia with 2% lidocaine. An incision along the sagittal midline from frontal bone to occipital bone was made and full thickness flap elevation was performed. Under the saline irrigation, four circular bi-cortical defects with a diameter of 8 mm were made by using a trephine bur. Then, randomly assigned graft materials were grafted into the defects. The flap was sutured with a biodegradable suture material (Monosyn, B-Braun, Melsungen, Germany) which was removed 10 days later. Five animals were sacrificed at 2 and 8 weeks of post surgery.

### Histologic processing

Before embedding in paraffin blocks, all sections were fixed using 10% buffered formalin solution (10% neutral buffered formalin, BBC Biochemical, Mount Vernon, WA, USA) and decalcified with 5% nitric acid (nitric acid, Daejung Chemicals & Metals Co., Kyonggi, Korea). Then, serial sectioning with 5 μm were performed along the sagittal direction of the rabbit calvarium. Two sections closest to the center of the defect were chosen and stained with hematoxylin and eosin and Goldner’s Masson trichrome.

### Analysis methods

#### Clinical and histologic observations

For the clinical observation, animals were carefully observed and evaluated for the presence of allergic reaction, exposure of graft materials and inflammatory reaction at 2 and 8 weeks after the surgery. For the histological observation, a binocular microscope (Leica DM LB, Leica Microsystems Ltd., Wetzlat, Germany) was used and all of the specimens were examined by a single blinded examiner.

#### Histomorphometric observations

**Figure 2:**
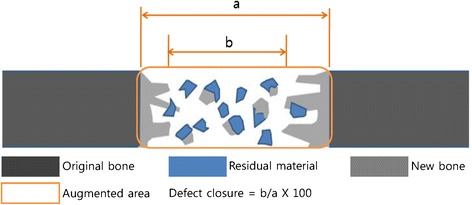
**Schematic diagram of histomorphometric analysis.**

For evaluating the biodegradation and bone healing, following four histomorphometric parameters were measured using an automated image-analysis system (Image-Pro Plus, Media Cybernetics, Silver Spring, MD, USA) (Figure 2).

1. New bone area: the area of newly formed bone in the defect.
2. Defect closure: the ratio of the distance between the newly formed bone over the initial distance between the original defect margins.
3. Augmented area: the area between the defect margins including bone tissues, marrow tissues, residual materials, fibrovascular tissues and adipose tissues.
4. Residual material: the area of remaining grafted materials in the defect.

#### Statistical analysis

All values were presented as mean ± standard deviation and a commercial software program (SPSS 21.0, SPSS Inc., Chicago, IL, USA) was used for the statistical analysis. For multiple comparisons among the groups in a given time, one-way ANOVA method test and Tukey-HSD post hoc procedure were performed. Independent *t*-test was carried out to analyze the statistical difference between 2 and 8 weeks healing period within each group.

## Results

### Clinical findings

All experimental sites showed uneventful healing during the postoperative healing period. Clinically, there was no specific evidence of complications such as exposure of graft materials.

### Histological findings

#### 2 weeks healing period

**Figure 3:**
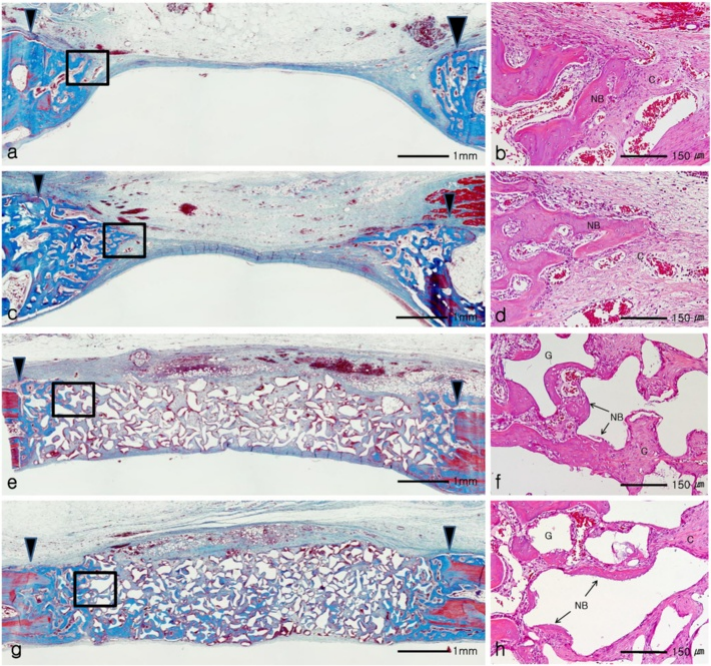
**Histologictransversal sections obtained 2 weeks after surgery: (a, b)** control group, **(c, d)** collagen sponge group, **(e, f)** BCP group, **(g, h)** BCPC group. Arrowheads = defect margin; NB = new bone; G = graft material; C = connective tissue. **(a, c, e, g)** Goldner’s Masson trichrome stain and original magnification: 40×; **(b, d, f, h)** Hematoxylin and eosin stain and original magnification: 200×.

All groups showed small amount of wedge shaped new bone formation limited to the areas of the defect margin and the amounts of newly formed bone seemed to be similar among all groups. In the control and collagen sponge group, the center of the defect was depressed and flattened by surrounding connective tissue and dura mater. In contrast, in BCP and BCPC group, the center of the defect was not depressed and the grafted materials maintained the space (Figure 3a, 3c, 3e, 3g).

On high magnification view, in the collagen sponge group, the grafted collagen sponge was resorbed and collapsed. In BCP and BCPC group, at the border of newly formed bone, osteoblasts and osteoids were found in close contact with BCP particles and inter-granular spaces were mainly filled with loose connective tissue. The collagen matrix of BCPC could not be clearly distinguished from the inter-particular connective tissues of BCP (Figure 3b, 3d, 3f, 3h).

#### 8 weeks healing period

**Figure 4:**
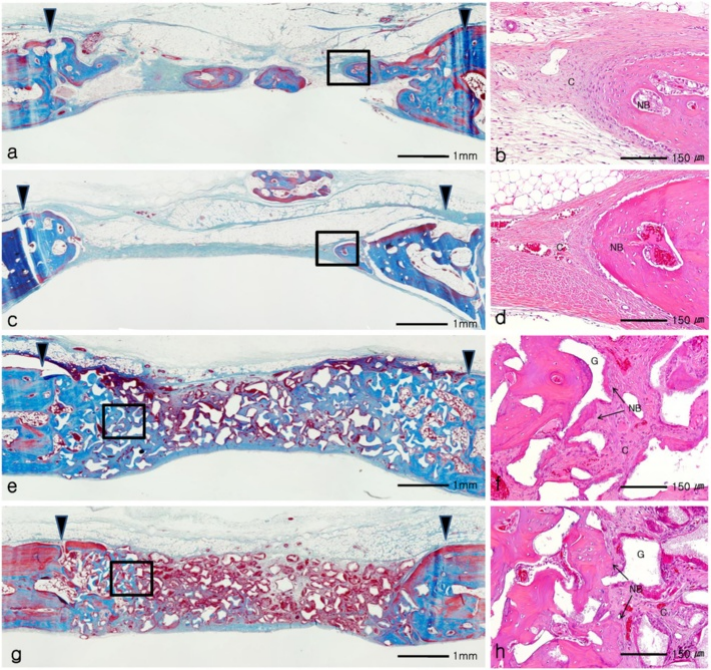
**Histologictransversal sections obtained 8 weeks after surgery: (a, b)** control group, **(c, d)** collagen sponge group, **(e, f)** BCP group, **(g, h)** BCPC group. Arrowheads = defect margin; NB = new bone; G = graft material; C = connective tissue. **(a, c, e, g)** Goldner’s Masson trichrome stain and original magnification: 40×; **(b, d, f, h)** Hematoxylin and eosin stain and original magnification: 200×.

The histological findings of the control and collagen sponge group were similar at 8 weeks healing period. In the control and collagen sponge group, more newly formed bone was found around the defect margin compared to those of 2 weeks healing period. However, the center of the defect was collapsed and mainly filled with loose connective tissue. Among a few specimens, the bony islands were found at the center of the defect (Figure 4a, 4b, 4c, 4d). In BCP and BCPC group, the sizes of BCP granules were similar between two groups, and reduced compared to those of 2 weeks healing period. The space between defect margins was maintained by grafted BCP particles. However, most of the newly formed bone was limited to the defect margin, and loose connective tissue and BCP particles occupied the space at the center of the defect (Figure 4e, 4f, 4g, 4h).

### Histomorphometric findings

The histomorphometric measurements are summarized in Tables 1, 2, 3 and 4. There was no statistically significant difference in new bone area and defect closure among all groups during the same healing period. All of the groups, except the collagen sponge group, showed statistically significiant increase of new bone formation between 2 and 8 weeks healing period. In terms of defect closure, the values significantly increased between 2 and 8 weeks healing periods in all of the groups (Tables 1, 2).

**Table 1:** **New bone area (mm** ^**2**^ **; Group Mean ± SD; n = 5)**

	**2 weeks**	**8 weeks**
**Control**	1.24 ± 0.35	3.29 ± 0.62^#^
**Collagen**	1.66 ± 0.85	2.43 ± 0.44
**BCP**	1.33 ± 0.49	2.47 ± 0.25^#^
**BCPC**	1.32 ± 0.56	2.41 ± 0.20^#^

There was no statistically significant difference among all groups in same healing period.

^#^Significantly different from same experimental group at 2 weeks.

**Table 2:** **Defect closure (%; Group Mean ± SD; n = 5)**

	**2 weeks**	**8 weeks**
**Control**	29.25 ± 4.61	54.76 ± 7.44^#^
**Collagen**	29.84 ± 5.97	48.78 ± 6.96^#^
**BCP**	26.29 ± 5.73	50.95 ± 3.66^#^
**BCPC**	26.52 ± 4.64	45.75 ± 11.62^#^

There was no statistically significant difference among all groups in same healing period.

^#^Significantly different from same experimental group at 2 weeks.

**Table 3:** **Augmented area (mm** ^**2**^ **; Group Mean ± SD; n = 5)**

	**2 weeks**	**8 weeks**
**Control**	4.37 ± 0.65	8.00 ± 0.79
**Collagen**	5.06 ± 2.34	5.59 ± 0.84
**BCP**	12.88 ± 2.45^#^	14.23 ± 0.71^#^
**BCPC**	14.35 ± 0.91^#^	12.92 ± 2.56^#^

^#^Significantly different from control and collagen group in same healing period.

**Table 4:** **Residual material (mm** ^**2**^ **; Group Mean ± SD; n = 5)**

	**2 weeks**	**8 weeks**
**Collagen**	0	0
**BCP**	3.89 ± 0.83	2.66 ± 0.43^#^
**BCPC**	4.24 ± 0.41	2.09 ± 0.63^#^

^#^Significantly different from same experimental group at 2 weeks.

The augmented area was statistically greater in BCP and BCPC group than those of the control and the collagen sponge group at 2 and 8 weeks healing periods. There was no statistically significant difference between 2 and 8 weeks healing periods in the same experimental group (Table 3).

The amount of residual material was similar between BCP and BCPC group at the same healing period and decreased between 2 and 8 weeks healing periods (Table 4).

## Discussion

Biologically, the optimal bone substitutes must possess biocompatibility, osteoconductivity, osteoinductivity and similar structure to human bone. They should provide structural framework for blood clot stabilization, maturation, and replacement space for newly formed bone [2,13]. In a clinical point of view, easy handling and cost-effectiveness are also important. Hence, many researches have been performed in a pursuit of both respects.

In the present study, we evaluated the osteoconductivity of BCPC in the rabbit calvarial defect. Nowadays the addition of collagen to bone substitute is expected to give benefits in bone regenerative therapy of dental field, on which many studies are focused. Collagen, which is one of the major organic components of human bone, has several advantageous properties for bone healing including hemostasis, chemotactic activity to attract fibroblast, and promotion of wound stabilization [14-16]. Moreover, it has been proven that collagen regulates gene expression and differentiation of osteoclasts [17]. Nonetheless, collagen itself has a major drawback as a bone substitute due to fast resorption by the activity of macrophages, leukocytes and bacteria, resulting in failure of space maintenance for new bone in-growth [15,18].

However, addition of collagen to bone substitute may compensate for the weaknesses of conventional bone substitutes. Particle type BCPs has been used in many studies and demonstrated space maintaining capacity and bone regenerative potential [8,19,20], but the performance of BCP on bone regeneration may be compromised in non-contained defects [9]. Block type BCP may be advantageous to overcome unfavorable defects, but its application may entail surgical difficulty and its three-dimensional microstructure may not be appropriate for bone in-growth [9,10]. With combination of BCP and collagen, BCP particles can provide volume stability and collagen matrix can promote cell proliferation and infiltration of osteoblasts [21].

Rabbit calvarial defect models have been used widely for evaluating newly developed bone substitutes because of adequate quantity and quality of bone marrow [22]. The critical size of rabbit calvarial defect has been considered as 10–15 mm through several studies [23-25]. Although 8 mm diameter rabbit calvarial defect is smaller than critical one, it has been known to be useful to evaluate defect healing pattern and bone regeneration [26,27]. And it has been proven that 8 mm circular defects in rabbit calvarium show limited bone healing within 8 weeks healing period in several studies [28,29]. Therefore, in this study, four 8 mm circular defects were made in the rabbit calvarium and bone healing was evaluated at 2 and 8 weeks after surgery, respectively. There could be a concern that extravasation of experimental materials from neighboring defects affected the results. However, the type of defect used in this study was contained. And no graft materials from another defect were found in the control site. Therefore the effect of neighboring defects can be thought to be minimal.

In histologic and histomorphmetric analysis, collagen sponge was totally resorbed and collapsed within 2 weeks healing period and showed similar healing pattern to control group. However, BCP and BCPC showed relatively slow resorption and maintained the space effectively, which is consistent with other previous studies [30,31]. This result suggests that both BCP and BCPC have favorable space maintaining capacity. The maintenance of the space for bone in-growth is important to stabilize blood clots and prevent downward growth of epithelium [32,33].

The amount of new bone and defect closure increased throughout the healing period in all groups. And there was no statistically significant difference among all groups at 2 and 8 weeks healing periods. Although BCP graft materials are commonly used as bone substitutes, the capacity of new bone formation of BCPs remains controversial. Numerous studies have demonstrated enhanced bone formation when BCPs were grafted in various defect models [7,29,30]. Whereas, some studies reported that grafted BCPs did not induce increased bone formation, which coincides with this study [31,34]. Therefore recent studies have tried to enhance the osteogenic capacity of BCP graft materials using osteoinductive materials such as BMPs [10,35]. Although increased new bone formation did not occur in this study, new bone was found in close contact with BCP particles in BCP and BCPC group with high magnification observation. This means that both BCP and BCPC are biocompatible and have good osteoconductive properties.

In this study, BCP and BCPC exhibited similar healing and bone regeneration pattern in total amount of new bone and augmented area. Both materials could be used as promising bone substitutes successfully. Further studies should be carried out to confirm that BCPC has advantages over BCP in the healing of non-contained type defects.

Collagen which is a component of BCPC has been widely used as a carrier of bioactive materials [36]. In a previous study, it has been shown that BCPC can be soaked with growth factors easily [37]. Therefore, BCPC could be also used as a promising carrier when bony defects are large or non-contained form where space maintenance becomes highly important.

## Conclusions

In conclusion, the BCPC and BCP demonstrated proper space maintaining capacity and osteoconductive property, suggesting BCPC can be efficiently utilized in various clinical situations.

## Availability of supporting data

The data sets supporting the results of this article are included within the article.
